# Interior design: how plant pathogens optimize their living conditions

**DOI:** 10.1111/nph.17024

**Published:** 2020-11-27

**Authors:** Claudia‐Nicole Meisrimler, Claudia Allan, Sophie Eccersall, Richard J Morris

**Affiliations:** ^1^ School of Biological Science University of Canterbury Private Bag 4800 Christchurch 8041 New Zealand; ^2^ Computational and Systems Biology John Innes Centre Norwich NR4 7UH UK

**Keywords:** abiotic stress, effector proteins, pathogens, plant immunity, signal transduction

## Abstract

Pathogens use effectors to suppress host defence mechanisms, promote the derivation of nutrients, and facilitate infection within the host plant. Much is now known about effectors that target biotic pathways, particularly those that interfere with plant innate immunity. By contrast, an understanding of how effectors manipulate nonimmunity pathways is only beginning to emerge. Here, we focus on exciting new insights into effectors that target abiotic stress adaptation pathways, tampering with key functions within the plant to promote colonization. We critically assess the role of various signalling agents in linking different pathways upon perturbation by pathogen effectors. Additionally, this review provides a summary of currently known bacterial, fungal, and oomycete pathogen effectors that induce biotic and abiotic stress responses in the plant, as a first step towards establishing a comprehensive picture for linking effector targets to pathogenic lifestyles.

## Introduction

To many microbes, plants are simply irresistible. They provide shelter, food, and water. More than that, plants offer flexible and adaptable living spaces with tuneable climate control and on‐demand supply of sugars and other nutrients for microbes to thrive and reproduce. Though some microbes are welcomed by the plant, such as symbionts, others, such as pathogens, need to be quite persistent to gain access to the type of all‐inclusive, reconfigurable accommodation that plants can supply.

How plants deter unwanted guests and manage their tenants has been reviewed extensively (Yu *et al*., 2017; Zhang *et al*., 2017; Liu *et al*., 2020). We will touch on these points only briefly and from the pathogen’s perspective. Our focus, here, is to review the latest results and findings on how pathogens manipulate plant processes to change the physical, chemical, and biological nature of their living spaces. The tools that pathogens use to make these alterations are diverse, with many still poorly characterized. Collectively, these host manipulation tools are known as effectors, typically small secreted proteins, metabolites, or RNA molecules (Büttner, 2016; Toruño *et al*., 2016). The importance of being able to interfere with their host is evident from the number of effectors in pathogen genomes. For example, it has been estimated that the hemibiotroph oomycete *Phytophthora infestans* has over 550 RxLR effector genes, and hemibiotroph bacteria like *Ralstonia*, *Pseudomonas* or *Xanthomonas* can reach from as few as three type III core effectors to as many as 32 (Haas *et al*., 2009; Roux *et al*., 2015; Büttner, 2016).

From the plant’s perspective environmental challenges are commonly divided into biotic and abiotic stress, and this has led to the identification of signalling pathways associated with the different types of stress adaptation. Biotic stress pathways detect pathogens, amongst other things, and launch a defence response, but plant–microbe interactions are not restricted to immune signalling (Velásquez *et al*., 2018; Saijo & Loo, 2020). Though many effectors have been reported to interfere with the plant’s immune system, there are examples of effectors targeting developmental pathways, such as the phytoplasma effectors that induce witches’ broom symptoms by degrading plant transcription factors involved in branching, leaf shape, and flower development (Chang *et al*., 2018; Wang *et al*., 2018). The resulting increased amount of vegetative tissue has been proposed to be beneficial for phytoplasma and their insect hosts (MacLean *et al*., 2014). Likewise, there is growing evidence that pathogens tweak other plant adaptation mechanisms to improve their cellular environment (Xin *et al*., 2016).

Here, we focus on the emerging field of the effectors’ role in perturbing abiotic stress pathways in plants. From the microbe’s perspective, biotic, abiotic, and developmental pathways all represent different routes to changing the environment the microbe is exposed to, such as the defence molecules that the plant produces, nutrients, other microbial populations, humidity, or the connectivity between cells. The separation into individual pathways results in effects that cannot be accounted for without considering ‘cross‐talk’ between modules; so, where appropriate, we point out such potential links.

## Taking up and leaving accommodation

To take up accommodation in a plant, a microbial pathogen needs to overcome pre‐existing physical barriers, such as lignified cell walls and a waxy cuticle. How pathogens gain access to plant cells depends on whether they need to keep those cells alive (biotrophic lifestyle) or not (necrotrophic lifestyle).

Necrotrophic pathogens have no hesitation in degrading their hosts and can take a destructive approach to entering. By contrast, biotrophs seek nondestructive colonization (Spoel *et al*., 2007; Wang *et al*., 2014). Stomata represent a natural access route into the host during the early infection stage and out of the host during the end of the life cycle. The latter is of particular importance for (obligate) biotrophic fungi and oomycetes that form conidiophores and sporangiophores for dispersion of their next generation. Secondary messengers and phytohormones regulating stomatal movement and water distribution in the plant, including abscisic acid (ABA), salicylic acid (SA), jasmonic acid (JA), ethylene (ET), and cytokinin need well‐organized manipulation to reach a desirable outcome for the pathogen (Su *et al*., 2017; Saijo & Loo, 2020). Interestingly, pathogens target plant abiotic stress adaptation pathways with so‐called ‘effectors’, though it is not clear if they do this in a targeted manner or if it is a by‐product of manipulating the host’s immune system.

## Effector proteins: tools of the trade

Pathogens have evolved a large set of tools (effectors) to ‘tweak’ the host’s machinery to suppress host defence, facilitate infection, and ultimately enhance the derivation of nutrients from the host (Büttner, 2016; Toruño *et al*., 2016). Effectors can be classified into two distinct groups: secreted effectors that act in the extracellular space of the host tissue (apoplastic space) and the translocated effectors that act within in the host’s cell (cytoplasmic space) (Büttner, 2016; Wang *et al*., 2017). To target key processes within a plant cell, the pathogen needs to find a way to translocate these effectors past the plant’s plasma membrane. Several bacterial pathogens use complex multi‐protein secretion systems, such as the type 3 and 4 Secretion System (T3SS, T4SS), which deliver an assortment of effectors into host cells (Costa *et al*., 2015). Filamentous pathogens, such as fungi and oomycetes, lack the bacterial secretion systems and employ a distinctly different, but far less understood, effector translocation method. For all their diversity, many effector proteins found in filamentous pathogens share common motifs. Characteristics of a variety of fungal (e.g. ToxA‐like, MAX, RxLR‐like, and RALPH) and oomycete (e.g. RxLR(‐like), crinkler, DEER, WY‐domain) effectors have been identified and many effectomes catalogued, yet much remains to be discovered and it will be of significant importance to understand the ‘when, where, and how’ filamentous pathogens translocate effector proteins in dependence of their lifestyle (Toruño *et al*., 2016; Wang *et al*., 2017; Wawra *et al*., 2017; Jones *et al*., 2018; Bozkurt & Kamoun, 2020). For pathogens, effectors are the key to success: they enable energy‐efficient manipulation of an existing system to live, feed, and prosper. In the following, we review recent insights into how pathogens make use of translocated effector proteins to manipulate the host’s cellular signal transduction mechanisms.

## How not to be seen

### The need for new disguises

If a pathogen is detected it can expect to be faced with a toxic chemical environment (reactive oxygen species (ROS), proteinases, chitinases, glucanases, etc.) as a consequence of the plant’s immune system doing its job (Yu *et al*., 2017). Plants detect microbes through microbe‐associated molecular patterns (MAMPs), which are perceived by specific pattern‐recognition receptors (PRRs) and trigger innate immune responses. MAMPs are highly conserved molecules or structural components, such as flagellin, that are indispensable for microbial fitness or lifestyle (Bigeard *et al*., 2015). PRRs are usually plasma‐membrane‐bound receptor‐like kinases or receptor‐like proteins with extracellular domains allowing MAMP perception (Boutrot & Zipfel, 2017). The signalling responses induced by PRR‐mediated perception of MAMPs is termed pattern‐triggered immunity (PTI). Recognition of these signals by PRRs leads to a rapid influx of cytosolic calcium ions (Ca^2+^), accumulation of apoplastic ROS, activation of mitogen‐activated protein kinases (MAPKs), regulation of phytohormones, upregulation of immunity‐associated gene expression, synthesis of antimicrobial proteins, and callose deposition (Bigeard *et al*., 2015). Pathogens would benefit from learning how not to be seen and have evolved effectors to interfere with a plant’s recognition of MAMPs and the downstream signal transduction pathways. As a countermeasure, plants have evolved a second mode of immunity: effector‐triggered immunity (ETI). Intracellular nucleotide‐binding site‐leucine‐rich repeat receptors can detect cellular damage exerted by effectors, upon which plant immunity is reinstated, involving also the hypersensitive response for targeted cell death at the site of infection (Lolle *et al*., 2020). Pathogens therefore have to continuously keep changing to not be recognized.

### Shooting the messenger

**Fig. 1:**
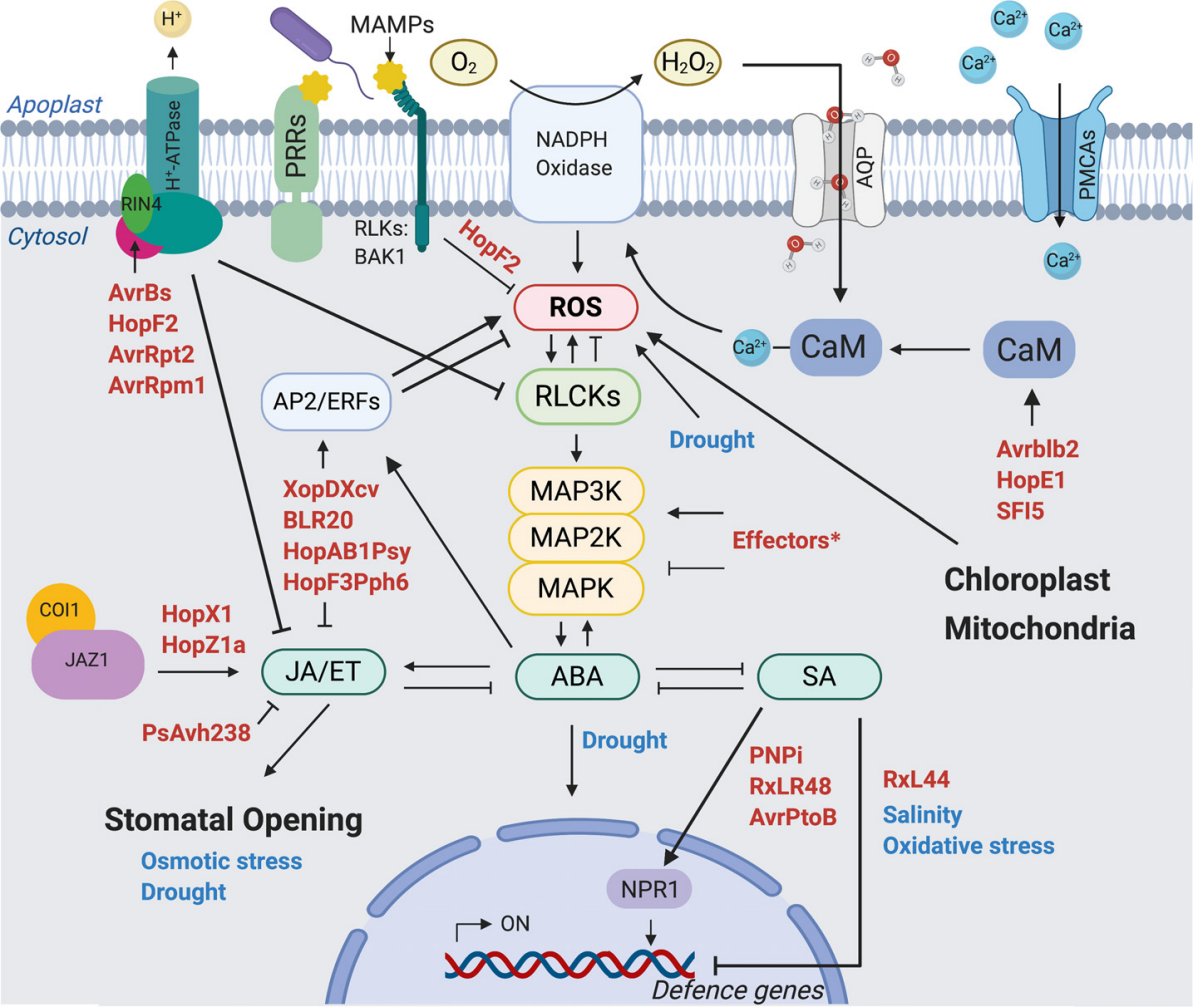
Overview of our present knowledge on effectors (red) targeting abiotic stress adaptation pathways in plant cells. RPM1‐interacting protein 4 (RIN4) is a target of *Phytophthora syringae* effectors, including AvrBs, AvrRpt2, AvrRpm1, and HopF2, inhibiting H^+^‐ATPase and downstream jasmonic acid (JA)/ethylene (ET) signalling. Avrblb, HopE1 and SFI5 target calmodulin (CaM), directly regulating Ca^2+^‐signalling. HopF2 interacts with BRI1‐associated kinase 1 (BAK1), inhibiting reactive oxygen species (ROS) production. A variety of effectors (Effectors*) are known to target mitogen‐activated protein kinase (MAPK) signalling. JA/ET hormonal signalling is manipulated by HopX1 and HopZ1a targeting the Jasmonate ZIM‐domain 1 (JAZ1) Coronatine‐insensitive protein 1 (COI) complex. Additionally, *Xanthomonas* spp. XopDXcv and lettuce downy mildew *Bremia lactucae* effector BLR20 inhibit JA/ET pathways by targeting AP2/ethylene response factor (ERF) transcription factors. PsAvh238 inhibits ET biosynthesis. Effectors from *Phytophthora capsici* (RxLR48), *Hyaloperonospora arabidopsidis* (RxL44), *Phytophthora syringae* (AvrPtoB) and *Puccinia striiformis* (PNPi) directly inhibit salicylic acid (SA) signalling for defence gene activation. HopAB1Psy and HopF3Pph6 interfere with ERF8, influencing abscisic acid (ABA) signalling. Lines ending with arrowheads indicate interactions, whereas lines ending with a short orthogonal line indicate inhibition. AQP, aquaporin; MAMPs, microbe‐associated molecular patterns; PCMAs, plasma membrane Ca^2+^‐ATPases; PPRs, pattern‐recognition receptors; RLKs, receptor‐like kinases.

If a pathogen cannot prevent its own detection by the plant, a first point of action is to develop strategies to disable the transmission of this information. This strategy includes targeting Ca pathways, MAPKs, and retrograde signalling. As phytohormones play prominent roles in plant defence they are frequently targeted by effectors (Büttner, 2016; Han & Kahmann, 2019). Interfering with these signalling pathways, however, has impacts on the plant beyond defence (Fig. 1; Supporting Information Table S1). Whether the associated changes to the plant are best viewed as collateral damage or specifically targeted manipulations by the pathogen to change plant properties to its advantage is, in many cases, not clear.

## Rewiring control systems

### Getting hormonal

As key signalling agents, phytohormones exert control over various plant processes, not just defence. Pathogens have learnt to manipulate such systems to their advantage. Effectors can alter concentrations of phytohormones and target proteins in hormone signal transduction pathways to facilitate the infection process. These topics have been well covered in recent reviews (Gimenez‐Ibanez *et al*., 2016; Toruño *et al*., 2016; Han & Kahmann, 2019), and we will touch them only superficially. Instead, we will focus on pathogens that use effectors to target the host machinery involved in abiotic stress adaptations (Fig. 1). The hormone pathways targeted by the pathogen depend on their lifestyle; for example, biotrophs will want to activate pathways that repress cell death, whereas necrotrophs will employ the opposite strategy (Spoel *et al*., 2007).

#### Salicylic acid, jasmonic acid and ethylene

In addition to its role in plant immunity, particularly against biotroph pathogens (Ding & Ding, 2020), SA has been shown to influence metabolism and gas exchange, which can contribute to reducing the negative impact of salt stress. As such, SA is involved in physiological processes such as photosynthesis and plant–water relations under stress conditions, and thereby provides protection for plants against abiotic stresses (Miura & Tada, 2014). The effector target NPR1 (Nonexpresser of PR genes 1) is a regulator of SA pathways linked to cold, salt, and oxidative stress tolerance, as well as plant immunity (Jayakannan *et al*., 2015; Saijo & Loo, 2020). A variety of effector proteins, including *Puccinia* NPR1 interactor (PNPi; *Puccinia striiformis*), RxLR48 (*Phytophthora capsici*), and AvrPtoB (*Pseudomonas syringae*) target NPR1 (Wang *et al*., 2016; Chen *et al*., 2017; Li *et al*., 2019). Potentially, NPR1 targeting might be important for bypassing PTI as much as for conditioning of the plant cell for pathogens by using osmotic stress adaptation processes.

The JA and ET signalling pathways have been shown to be essential for defence against herbivores, insects, and necrotrophic pathogens (Broekgaarden *et al*., 2015). Similar to NPR1, JAZ (Jasmonate‐Zim‐domain) proteins play a role in abiotic stress adaptation processes; for example, JAZ3 is involved in salt stress adaptation processes (Valenzuela *et al*., 2016). Several pathogenic microbes use effector proteins to modify JAZ proteins in a direct and indirect manner. This includes the *Pseudomonas* effectors HopX1, HopZ1a, AvrRpm1, AvrRpt2 and AvrB and the *Hyaloperonospora arabidopsidis* effector RxL44 (Caillaud *et al*., 2013; Gimenez‐Ibanez *et al*., 2016; Ma & Ma, 2016; Büttner, 2016). ET pathways are also targeted by effectors, for example *Phytophthora sojae* has been shown to use PsAvh238 to suppress ET biosynthesis and facilitates infection (Yang *et al*., 2019), and *P. syringae* uses HopAF1 to target methionine recycling that disrupts ET biosynthesis (Washington *et al*., 2016). In contrast to the established concept of antagonism between the ‘immunity’ hormone SA and JA/ET pathways in response to pathogens with a specific lifestyle, recent results have expanded this network to include ABA, thereby starting to reveal how abiotic stress adaptation and plant immunity are integrated.

#### Abscisic acid

ABA has long been associated with abiotic stress responses, but in recent years a role in plant immunity has also been demonstrated (Han & Kahmann, 2019). In particular, the role of ABA in stomatal opening and closure is of interest for most pathogens, a topic we will revisit later.

ABA has been shown to both reduce and enhance resistance to *Pseudomonas*, depending on the stage of infection. For example, *Pseudomonas* uses the effector HopF2 (a mono‐ADP‐ribosyltransferase) to inhibit kinase activity of MKK5, MPK4 and MPK6 (Wang *et al*., 2010; Wu *et al*., 2011) by interacting with PUB35 (a U box domain‐containing E3 ubiquitin ligase) and the kinase SNRK3.22 – a negative regulator of ABA responses (Lumba *et al*., 2014; Cao *et al*., 2019). On the other hand, expression of *Pseudomonas* effector AvrPtoB in Arabidopsis led to elevated ABA levels (de Torres‐Zabala *et al*., 2007; Cheng *et al*., 2011; Wang *et al*., 2019). Also, AvrPtoB is known to interact with NPR1, EXO70B1 (an exocyst complex component), BAK1 (Brassinosteroid Insensitive 1‐associated receptor kinase 1, a transmembrane kinase that associates with receptors such as BRI1 during brassinosteroid signalling or with EFR and FLS2 to signal the recognition of MAMPs), and FLS2 (leucine‐rich repeat receptor‐like serine/threonine‐protein kinase FLS2; specific flg22 recognition). By increasing ABA levels, *Pseudomonas* seems to antagonize the SA pathway required for resistance to this pathogen during the first stage of the infection. Recently, it has been shown that a variety of pathogens target members of the APETALA2/ET response factor (AP2/ERF) transcription factor family, involved in ET and ABA‐mediated signalling. *Xanthomonas* spp. (XopDXcv), *Pseudomonas* (HopF3Pph6, HopAB1Psy), and lettuce downy mildew *Bremia lactucae* (BLR20) have been reported to target AtERF4 (Arabidopsis), AtERF8 (Arabidopsis), and LsERF093 (closest AtERF5 lettuce orthologue), respectively (Büttner, 2016; Cao *et al*., 2019; Pelgrom *et al*., 2020). Interestingly, ERF4 and ERF8 possess an EAR motif that facilitates transcriptional repression instead of activation of downstream genes with roles in osmotic and drought stress adaptation (Xie *et al*., 2019). Many abiotic stresses lead to plant responses via an increase of ABA levels (in particular, desiccation, cold, and osmotic stress); and though existing data show that pathogens target ABA signalling pathways, it is not clear if this targeting aims to directly invoke abiotic, ABA‐induced responses or to indirectly modulate SA, JA/ET signalling to weaken plant immunity, or both. In this context, much remains to be learnt about the temporal link of the pathogen life cycles, effector secretion/translocation, and modification of the ABA signalling pathway.

#### Auxins, cytokinins and brassinosteroids

These phytohormones are often associated with growth and development, but also with biotic and abiotic stress. There is now ample evidence to demonstrate that all these hormone pathways are targeted by effectors (Han & Kahmann, 2019). For instance, it has been shown that the *P. syringae* effector AvrRpt2 targets auxin and enhances its turnover, HopQ1 activates cytokinin signalling, and AvrPto and AvrPtoB target brassinosteroid signalling via BAK1, which interacts with the brassinosteroid receptor BRI1 (Shan *et al*., 2008; Büttner, 2016). The mechanisms of these effectors remain poorly understood, however. Thus, much is left to be discovered, even in the well‐studied area of plant hormone signalling, in terms of what pathogens gain from their manipulation that enhances their lifestyle.

### Interfering with calcium signalling

Ca^2+^ plays a key role as a secondary messenger in diverse plant processes (Dodd *et al*., 2010). Calmodulin (CaM) is one of the most extensively studied Ca^2+^‐sensing proteins and has been shown to be involved in transduction of Ca^2+^ signals in response to abiotic and biotic stress (Wilkins *et al*., 2016; Tian *et al*., 2019). Thus, the finding that effectors can target CaMs permits the speculation that pathogens have the potential to directly modulate the Ca^2+^ sensing machinery in plants. Though clear immediate targets of such interactions relate to immune signalling by HopE1 from *P. syringae* (Guo *et al*., 2016) and SFI5 and Avrblb2 from *P. infestans* (Zheng *et al*., 2018; Naveed *et al*., 2019), it will be fascinating to uncover to what extent Ca^2+^ signalling in other contexts is exploited by pathogens. A candidate for the integration between different pathways is the NAC transcription factor NTL9, which is a Ca^2+^‐dependent CaM binding protein that acts as a suppressor of transcription. NTL9 is induced by osmotic stress, and its transcript levels are also enhanced by SA, drought, and heat stress. The effector HopD1 of *P. syringae* has been shown to interact with NTL9 to suppress ETI (Block *et al*., 2014). Given involvement of NTL9 in both biotic and abiotic stress, one might expect that its targeting by HopD1 will disrupt several pathways. A further example comes from the analysis of the effector repertoire of the biotrophic fungus *Microbotryum lychnidis‐dioicae* that identified a small secreted protein that interacts with a Ca^2+^‐dependent lipid binding protein in Arabidopsis (AtCLB; Kuppireddy *et al*., 2017). AtCLB has been shown to negatively regulate the enzyme Thalional synthase 1, which is involved in the response to drought. The same effector potentially targets cellulose synthase interactive protein 1, which also contains a putative Ca^2+^‐binding domain and has been shown to be important for the stability of microtubules. In both cases, the effector binds to the potential Ca^2+^‐binding domain, leading to a potential modulation of the signalling pathways for the benefit of the fungus (Kuppireddy *et al*., 2017).

### Making radical changes

In plants, ROS (most commonly superoxide ion (O_2_^•^^−^), hydrogen peroxide (H_2_O_2_), and hydroxyl ion radical (OH^•^) are associated with a wide variety of physiological processes. ROS have versatile functions; for example, they are involved in ETI and hypersensitive response (inducing local cell death), but they also have a nondestructive function as signalling molecules in abiotic stress adaptation, as well as in PTI (Leister, 2012; Balint‐Kurti, 2019; Kretschmer *et al*., 2019).

Electron transport chains in chloroplasts and mitochondria are major sites of ROS production due to potential premature electron leakage to oxygen and appear to be integrated into the plant immune system and abiotic stress adaption (Clercq *et al*., 2013; Ng *et al*., 2013; Kretschmer *et al*., 2019). The ability to modify the host’s ROS system can mean death or life for a pathogen (Jwa & Hwang, 2017). Evidence lets us hypothesize that necrotrophic pathogens increase the plant's ROS production to induce cell death; for example, the necrotrophic wheat pathogen *Pyrenophora tritici‐repentis* produces ToxA in a host‐selective manner. ToxA interacts with the wheat chloroplast ToxA Binding Protein 1 and with the Arabidopsis homologue Thylakoid formation 1, inducing cell death in a light‐dependent manner by increased ROS accumulation in chloroplasts (Kretschmer *et al*., 2019). Biotrophic and hemibiotrophic pathogens seem to associate with a significantly different strategy by trying to reduce ROS production. The hemibiotroph bacterium *Pantoea stewartii*, a wilt and leaf blight pathogen of maize, translocates the effector WtsE (a member of the AvrE family) with a major impact on secondary metabolism (phenylpropanoid pathways) and suppression of photosynthesis‐related gene transcription (Asselin *et al*., 2015). *Puccinia striiformis*, an obligate biotrophic wheat stripe rust fungus, translocates Pst_12806 into the host, where it targets the chloroplast (Xu *et al*., 2019). Xu *et al*. (2019) confirmed that Pst_12806 interacts with the C‐terminal Rieske domain of TaISP (putative component of the cytochrome *b*_6_ *f* complex). In contrast to ToxA, Pst_12806 expression *in planta* reduced the electron transport rate, photosynthesis, and production of ROS in the chloroplasts. These examples indicate that ROS modulation is associated with the lifestyle of a particular pathogen and how it interacts with the host.

### Perturbing retrograde signalling

**Fig. 2:**
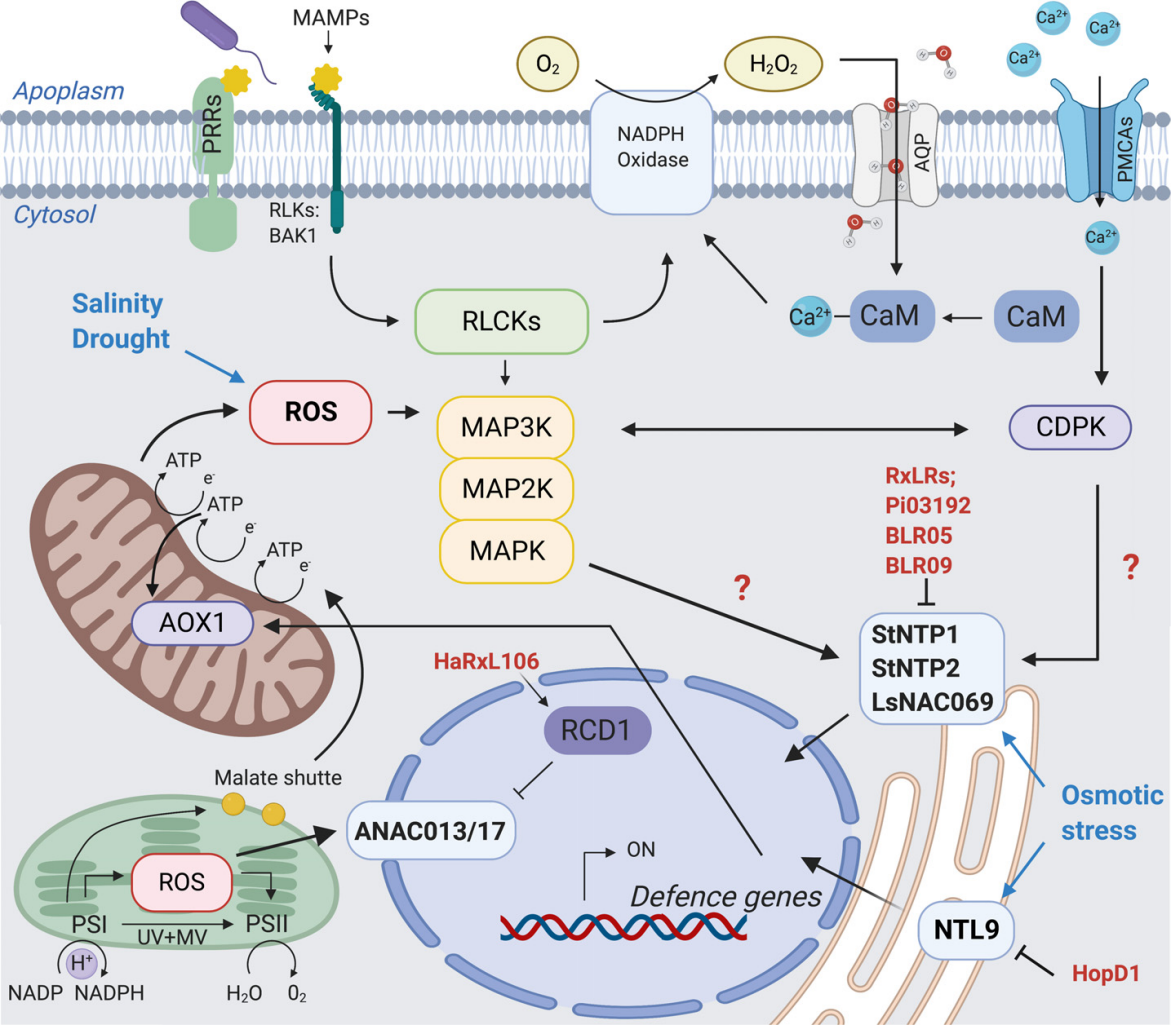
Effectors target components of retrograde signalling. Arabidopsis radical‐induced cell death1 (RCD1) is a target of the *Hyaloperonospora parabidopsidis* effector HaRxL106, promoting interplay between RCD1 and NAC transcription factor ANAC013/17. The *Phytophthora infestans* RxLR effector Pi03192 targets the NAC transcription factor StNTP1 and StNTP2, and the *Bremia lactucae* effectors BLR05 and BLR09 target the NAC transcription factor LsNAC069, inhibiting translocation of these potato and lettuce orthologues of ANAC013/17 from the endoplasmic reticulum to the nucleus, promoting disease. ANAC013/17 translocation is induced upon increased reactive oxygen species (ROS) production in chloroplasts and mitochondria, leading to transcriptional activation of downstream targets. Osmotic stress induces NAC transcription factor NTL9 translocation, which is also a direct target of HopD1 (inhibiting effector triggered immunity (ETI)). Solid ended arrows indicate interactions, whereas blunt end arrows indicate inhibition. Question marks indicate hypothesized interactions. AQP, aquaporin; CDPK, calcium‐dependent protein kinase; MAMPs, microbe‐associated molecular patterns; MV, methyl viologen; PCMAs, plasma membrane Ca^2+^‐ATPases; PPRs, pattern‐recognition receptors; PSI/II, photosystem I/II; RLCKs, receptor‐like cytoplasmic kinases; RLKs, receptor‐like kinases; ROS, reactive oxygen species; UV, ultraviolet light.

In terms of disrupting signalling processes in the plant, a key target for biotroph and hemibiotroph pathogens is retrograde signalling. Retrograde signalling involves signals, for example ROS, which are generated in the chloroplasts and mitochondria, exported from the organelles through the cytosol to the nucleus where they modulate gene expression (Leister, 2012). It has been discovered that NAC transcription factors ANAC013 and ANAC017 have important roles in ROS‐associated retrograde signalling (yet to be confirmed for the orthologues in potato (StNTP1 and StNTP2) and lettuce (LsNAC069)). ANA0C17 is a positive regulator of mitochondrial alternative oxidase 1a, a key player in mitochondrial ROS scavenging (Ng *et al*., 2013). And ANAC013 mediates mitochondrial retrograde regulation induced expression of mitochondrial dysfunction stimulon (MDS) genes, which affect mitochondrial functions and significantly influence ROS production and redox status of chloroplasts (DeClerq *et al*., 2013; Shapiguzov *et al*., 2019). In particular, biotroph and hemibiotroph oomycetes seem to target the retrograde signal associated with these NAC transcription factors. All effectors identified so far (Pi03192, BLR05 and BLR09), co‐localize with their targets StNTP1, StNTP2 and LsNAC069 in the endoplasmic reticulum upon expression *in planta*, where they inhibit PTI‐induced translocation of the host proteins to the nucleus (Fig. 2; McLellan *et al*., 2013; Meisrimler *et al*., 2019). Interestingly, in lettuce, LsNAC069 translocation was also induced by osmotic stress, which was similar to PTI‐induced translocation inhibited by the effector proteins BLR05 and BLR09. Generally, NAC transcription factors have been reported to be involved in drought and osmotic stress resilience associated with the ABA‐independent and ROS‐dependent signalling pathway (Nakashima *et al*., 2014). Furthermore, Radical‐induced Cell Death 1 (RCD1), a direct interactor of ANAC013 and ANAC017, is also targeted by the *H. arabidopsidis* effector HaRxL106 (Wirthmueller *et al*., 2018; Shapiguzov *et al*., 2019). Arabidopsis HaRxL106 overexpression lines showed a shade avoidance phenotype with SA levels comparable to wild‐type plants but attenuation of the transcriptional activation of SA‐induced defence genes (including the NPR1‐dependent pathway). Furthermore, these lines partially overlapped in their transcription profile with the *rcd1*‐deficient mutant, which shows enhanced sensitivity to apoplastic ROS and salt stress but increases tolerance to chloroplastic ROS related to MDS gene expression regulated by ANAC013 and ANAC017 (Fig. 2). During the biotroph life stage, pathogens appear to rely on a healthy balance of retrograde and ROS signalling. But is that because they want to stay hidden or because they need to maintain chloroplast and mitochondria function during the infection process?

## Adjusting the water supply

**Fig. 3:**
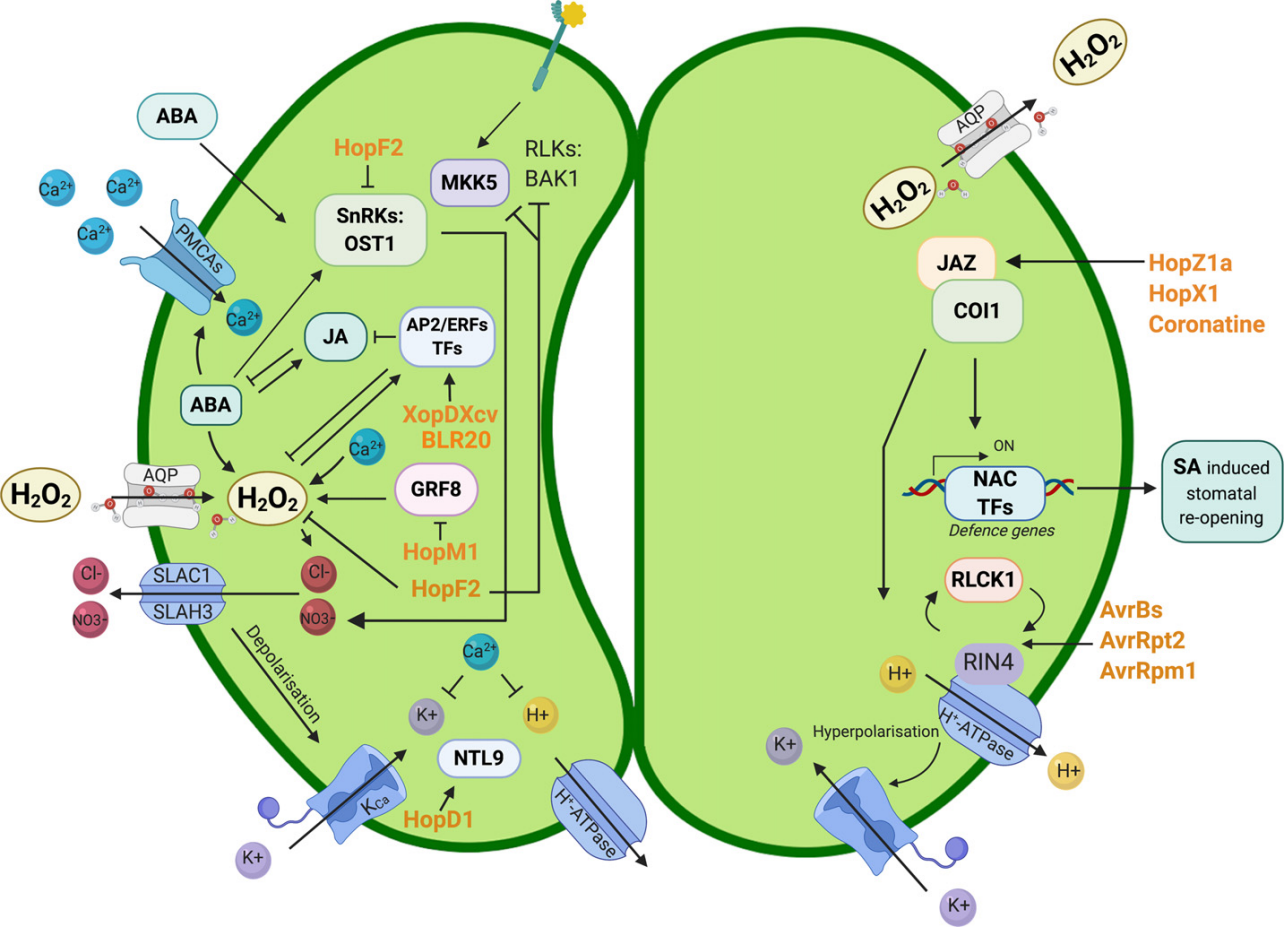
Effectors can induce changes in stomatal aperture before and during infection, which may affect drought, osmotic, high light, and other stress adaptation processes. HopF2, AvrRpm1, AvrRpt2 and AvrBs induce stomatal closure. XopDXcv and BLR20 inhibit jasmonic acid (JA) and abscisic acid (ABA) signalling. HopM1 downregulates growth regulating factor 8 (GRF8), inhibiting reactive oxygen species (ROS) production. Similarly, HopF2 inhibits ROS production via BRI1‐associated kinase 1 (BAK1), Mitogen‐activated protein kinase kinase 5 (MKK5) and RPM1‐interacting protein 4 (RIN4). Hopz1a and HopX1 directly target Jasmonate ZIM‐domain (JAZ) proteins, hindering stomatal closure, in addition to interacting with its receptor F‐box protein Coronatine‐insensitive protein 1 (COI1) to induce stomatal reopening. Coronatine also targets JAZ for reopening of stomata. HopD1 interferes with the NTM1‐like protein 9 (NTL9) pathway, which is triggered by flg22‐induced pattern triggered immunity (PTI) and during stomatal closure. Many of the effectors shown result in a decrease in resistance in plants, leading to increased infection by bacteria and distribution of spores during the end of the fungal and oomycete life cycle. Effectors are shown in orange. Lines ending with arrowheads indicate interactions, whereas lines ending with a short orthogonal line indicate inhibition. AP2/ERFs, APETALA2/ethylene response factors; AQP, aquaporin; RLCK1, receptor‐like cytoplasmic kinase 1; RLKs, receptor‐like kinases; SA, salicylic acid; SLAC1, Slow Anion Channel‐associated 1; SLAH3, SLAC1 homologue 3; SnRKs, SNF‐related serine/threonine‐protein kinases; TFs, transcription factors.

Once pathogens have gained access and bypassed the plant’s defence systems, they can focus on tweaking a range of other systems to their liking (Fig. 1). Many airborne pathogens manipulate physiological processes in plants associated with water homeostasis. One such process is the opening and closing of stomata, which is directly associated with the plant's water acquisition and distribution (Fig. 3; Melotto *et al*., 2006; Su *et al*., 2017). Pathogen transmission and infection are enhanced in conditions of rain, high air humidity, and high soil moisture, particularly those that infect aerial tissues (Xin *et al*., 2016; Velásquez *et al*., 2018). Pathogens have been shown to extensively target the SWEET sugar transporter family involved in water homeostasis by a variety of transcription activator‐like effectors (Chen, 2014). Weßling *et al*., 2014 reported in their supplemental information that the NIP1‐1, a plasma membrane aquaporin, interacts with the effector HopD1 group; furthermore, *Pseudomonas* AvrE and HopM1 are involved in the manipulation of water homeostasis (Weßling *et al*., 2014; Xin *et al*., 2016; Table S1). Water seems to be a driving factor for pathogens to successfully establish on the host, as much as it is a key factor for the survival of the plant and connects a variety of abiotic stress responses with plant immunity (Fig. 1; El Kasmi *et al*., 2018; Bulgakov *et al*., 2019; Saijo & Loo, 2020).

## Stomatal movement: an example of biotic and abiotic convergence

As an example for how effectors reveal the interconnectedness of biotic and abiotic adaptation pathways, we discuss stomatal movement. Stomata open and close to regulate gas exchange and water resources. They also present an access route into the plant for pathogens. Upon detection of MAMPs, stomata close to limit access, a process that is countered by effectors. ABA is part of the regulatory pathway responsible for stomata closure and opening in response to changes in water status, but it also initiates closure upon pathogen attack (Fig. 1; Xu *et al*., 2013; Cao *et al*., 2019). Flg22‐triggered PTI signalling congregates at the SNF‐related serine/threonine‐protein kinase Open Stomata 1 (OST1), which influences downstream activation of Slow Anion Channel‐associated 1 and 3 (SLAC1, SLAC3) and ABA signalling (Deger *et al*., 2015). OST1 regulates stomatal closure and may represent a target of the effector HopF2 to counter this defence response (Hurley *et al*., 2014). Moreover, ABA‐induced stomatal closure is closely correlated with Ca^2+^ and ROS waves. ROS generation, such as H_2_O_2_, is reliant on RbohD and OST1‐dependent phosphorylation of RbohF (Kimura *et al*., 2012). Overexpression of HopF2 in plants inhibits ROS production, stopping PAMP‐induced stomatal closure (Fig. 3; Hurley *et al*., 2014). Similarly, HopM1 downregulates growth regulating factor 8, in turn inhibiting ROS production. Interestingly, the fungal phytotoxic effector fusicoccin initiates an interaction between the 14‐3‐3 protein and the plasma membrane H^+^‐ATPase, fuelling stomatal opening, observably due to a disparate lifestyle aimed to gain hyphal entry into the cytoplasm (Camoni *et al*., 2018). Multiple *P. syringae* effectors AvrBs, HopF2, AvrRpt2, and AvrRpm1 target stomatal aperture, also influencing drought‐stress pathways (Melotto *et al*., 2006, 2017; Ray *et al*., 2019). AvrB specifically targets RIN4, initiating accumulation of receptor‐like cytoplasmic kinase 1 in turn, phosphorylating RIN4 and leading to wider stomatal aperture. One effector in particular, the toxin coronatine involved in *P. syringae* pv tomato DC3000 infection, directly implicates drought stress pathways by reopening stomata. Interestingly, Lee *et al*. (2015) demonstrate that *ripk* and *rin4* knockout lines are insensitive to coronatine‐induced stomatal reopening (Lee *et al*., 2015). Furthermore, *P. syringae* uses the translocated effector Hopz1a and HopX1 to directly target JAZ proteins, as well as interacting with its receptor Coronatine‐insensitive protein 1 (COI1) and thus hindering stomatal closure (Jiang *et al*., 2013; Gimenez‐Ibanez *et al*., 2014). COI1‐mediated JAZ2 (a coronatine target) degradation has been shown to activate MYC2, MYC3, and MYC4 to directly regulate the expression of *ANAC19*, *ANAC55*, and *ANAC72* to modulate stomatal aperture (Fig. 3; Gimenez‐Ibanez *et al*., 2016). As mentioned earlier, *P. syringae* uses HopD1 to interfere with the NTL9 signal transduction pathway, which is activated by flg22‐triggered PTI and stomatal closure (Block *et al*., 2014). NTL9 is mainly expressed in guard cells and has been shown to mediate osmotic stress signalling in leaf senescence and functions synergistically with Suppressor of Npr1‐1, Inducible 1 as negative regulator of pathogen‐induced PR1 expression (Kim *et al*., 2012). NAC transcription factors have gained attention in recent years, and emerging results suggest that they play a key role in co‐regulation of plant immunity, drought, osmotic stress, and senescence (Yuan *et al*., 2019).

## Conclusions

By definition, plant responses to pathogens should fall within biotic stress adaptation pathways. However, it is becoming increasingly apparent that this modularization and classification of pathways based on defined stresses can be misleading. From the microbe’s view, these plant pathways are all merely means by which the microbe can manipulate its environmental parameters. Many signalling agents are common to multiple modules, and perturbations to shared components can have knock‐on effects on seemingly unrelated responses. This is particularly striking for the case of immune responses in guard cells (Fig. 3). Closure of stomata may be a useful response to the biotic challenge of pathogens, but this response leads immediately to the induction of abiotic responses relating to gas exchange, water, cooling, and photosynthesis.

Major challenges for future research in the area will be to determine what parameters of the plant the pathogen is trying to manipulate, the mode of action of effectors, and their collateral damage caused by interdependencies of many pathways in plants. These challenges will likely have different answers depending on the lifestyle of the pathogen (Spoel *et al*., 2007). Understanding what processes effectors are targeting and with what aim (i.e. how this benefits the pathogen’s lifestyle choices), will be key for an integrated view of plant–microbe interaction strategies (Box 1).

Higher resolution data are often coupled to technological advances. In addition to established techniques already employed with success to study plant–microbe interactions, there are a number of exciting new technological developments that may help gain further insights (Nobori *et al*., 2018). These include the steady advances in live cell imaging and, in particular, new molecular probes and sensors, spatio‐temporal gene expression analysis, metabolomics, and developments in data analysis such as deep learning and causal inference. We look forward to the exciting results emerging from such studies and the holistic view of effector mechanism and function that will be gained (Box 1).

Box 1 **Future research questions.**

- How do effectors interfere with plant signalling and what information is being lost/given/distorted?
- How can we disentangle the action of effectors from the counter response of the plant?
- How does the mode of action of effectors differ between pathogen lifestyles?
- How can we use effectors as tools to learn more about plants and, in particular, the interdependencies of different regulatory pathways?
- How does stress in the plant affect effector expression in pathogens?
- How can we use effector proteins to specifically target pathways that will improve climate change resilience (and both biotic and abiotic consequences thereof)?

## Author contributions

C‐NM conceived the review in discussion with RJM. C‐NM led on manuscript preparation. C‐NM and RJM wrote the manuscript with contributions from SE and CA. CA designed the figures. All authors have read and approved the final version.

## Supporting information


**Table S1** Effector proteins and their potential abiotic stress targets in plants.
Please note: Wiley Blackwell are not responsible for the content or functionality of any Supporting Information supplied by the authors. Any queries (other than missing material) should be directed to the *New Phytologist* Central Office.
